# Clinical impact of the type VI secretion system on virulence of *Campylobacter* species during infection

**DOI:** 10.1186/s12879-019-3858-x

**Published:** 2019-03-07

**Authors:** Jessica Agnetti, Helena M. B. Seth-Smith, Sebastian Ursich, Josiane Reist, Marek Basler, Christian Nickel, Stefano Bassetti, Nicole Ritz, Sarah Tschudin-Sutter, Adrian Egli

**Affiliations:** 1 Applied Microbiology Research, Department of Biomedicine, University of Basel, Basel, Switzerland; 2 Clinical Microbiology, University Hospital Basel, Petersgraben 4, 4031 Basel, Switzerland; 3 Biozentrum, University of Basel, Basel, Switzerland; 4 Current address: Institute for Molecular Cancer Research IMCR, University of Zürich, Zürich, Switzerland; 5 Emergency Department, University Hospital Basel, Basel, Switzerland; 6 Department of Internal Medicine, University Hospital Basel, Basel, Switzerland; 7 Paediatric Infectious Diseases and Vaccinology, University Children Hospital Basel, Basel, Switzerland; 8 Department of Pediatrics, Royal Children’s Hospital Melbourne, University of Melbourne, Parkville, Australia; 9 Infectious Diseases and Hospital Epidemiology, University Hospital Basel, Basel, Switzerland

**Keywords:** Type IV secretion system, *Campylobacter jejuni*, *Campylobacter coli*, Virulence, Diarrhoea, Infection, Clinical course

## Abstract

**Background:**

The clinical course of *Campylobacter* infection varies in symptoms and severity depending on host factors, virulence of the pathogen and initiated therapy. The type VI secretion system (T6SS) has been identified as a novel virulence factor, which mediates contact-dependent injection of enzymes and toxins into competing bacteria or host cells and facilitates the colonisation of a host organism. We aimed to compare the clinical course of *Campylobacter* infection caused by strains with and without the T6SS and identify possible associations between this putative virulence factor and the clinical manifestations of disease.

**Methods:**

From April 2015 to January 2017, patients with detection of *Campylobacter* spp. were identified at the University Hospital of Basel and the University Children’s Hospital of Basel and included in this case-control study. Presence of the T6SS gene cluster was assayed by PCR targeting the *hcp* gene, confirmed with whole genome sequencing. Pertinent clinical data was collected by medical record review. Differences in disease- and host-characteristics between T6SS-positive (case) and –negative (control) were compared in a uni- and multi-variable analysis. Hospital admission, antibiotic therapy, admission to intensive care unit, development of bacteraemia and in-hospital mortality were considered as clinical endpoints.

**Results:**

We identified 138 cases of *Campylobacter jejuni* infections and 18 cases of *Campylobacter coli* infections from a paediatric and adult population. Analyses were focused on adult patients with *C. jejuni* (*n* = 119) of which 16.8% were T6SS-positive. Comparisons between T6SS-positive and -negative *C. jejuni* isolates did not reveal significant differences regarding clinical manifestations or course of disease. All clinical endpoints showed a similar distribution in both groups. A higher score in the Charlson Comorbidity Index was associated with T6SS-positive *C. jejuni* isolates (*p* < 0.001) and patients were more likely to have a solid organ transplant and to be under immunosuppressive therapy.

**Conclusions:**

Our study does not provide evidence that T6SS is associated with a more severe clinical course. Interestingly, T6SS-positive isolates are more commonly found in immunocompromised patients: an observation which merits further investigation.

**Electronic supplementary material:**

The online version of this article (10.1186/s12879-019-3858-x) contains supplementary material, which is available to authorized users.

## Background

Infection with *Campylobacter* spp. is a worldwide leading cause of diarrhoea, showing an increase in incidence over the last decade in North America, Europe and Australia [1]. In Switzerland, as in other European countries, campylobacteriosis is currently the most commonly reported food-borne bacterial disease [2] with up to 70% of all cases attributable to chicken consumption [3], thus posing a relevant public health problem. In Switzerland, the incidence in 2017 was 85.4 cases per 100,000 people [4] and the annual economic burden due to laboratory-confirmed campylobacteriosis has recently been estimated at around €8.3 million [5]. It is assumed that a large number of additional cases go unreported [5].

The clinical course of campylobacteriosis varies in symptoms and severity. Infection with *Campylobacter jejuni* - the most frequently diagnosed species - is most often characterised by acute diarrhoea, fever, and abdominal pain [6]. In most cases self-limiting, symptoms typically last from one day to a week, however, may persist for longer in 10–20% of patients and relapse may occur in another 5–10% [6]. The disease is occasionally further complicated by bacteraemia and sepsis or by extra-gastrointestinal manifestations following enteric disease, such as reactive arthritis and Guillain-Barré syndrome [6]. Complications and prolonged disease usually affect elderly or immunocompromised patients with comorbidities [1].

The clinical manifestation of infection with *Campylobacter* spp. is determined by host factors such as immune-competency, age, genetic background, and bacterial factors such as virulence of the pathogen, initial infectious dose, and initiated therapy [6, 7]. Current research has identified novel mechanisms that may contribute to virulence and survival of *Campylobacter* spp. in humans, such as the Type VI Secretion System (T6SS) [8], one of several bacterial systems for protein secretion [9]. With a structure similar to the tail of the contractile bacteriophages [10], the T6SS allows the contact-dependent secretion of effectors into other bacterial or host cells [11, 12]. The T6SS is encoded by a gene cluster of approx.17kb comprising 13 genes [13]. One of the hallmark genes of the T6SS gene cluster is *hcp* (encoding the secreted component hemolysin co-regulated protein) [14], which has been defined as a marker of a complete T6SS [15].

The expression of T6SS in *C. jejuni* has been demonstrated to be of importance for host cell adhesion and invasion in vitro: mutants with a defective secretion system show an up to 50% reduced ability to adhere to and invade colonic epithelial cells and macrophages [13]. Furthermore, in vivo studies show a higher persistence of *C. jejuni* expressing T6SS, with the dynamics of T6SS-regulation favouring colonisation of the colon [12, 13]. In polymicrobial environments such as the gastrointestinal tract, the T6SS also presents an advantage by allowing the pathogen to overcome the local microbiota [16].

In view of the diverse functionality of T6SS and extensive in vitro/in vivo research, the secretion system may provide a survival benefit for *Campylobacter* spp. contributing to successful and persistent infection [17]. A possible connection between T6SS as a clinically relevant virulence factor in *Campylobacter* spp. and manifestation of disease in humans has been insufficiently studied so far. Harrison et al. [15] found a correlation between T6SS-positive *C. jejuni* strains and bloody diarrhoea, and accordingly more severe disease, in a sample of 36 patients from Vietnam. A causal relationship could, however, not be proven [15].

In this study, we aimed to compare the clinical course of *Campylobacter* infection caused by strains with and without T6SS in an observational case-control study, and to identify possible associations between this virulence factor and the clinical manifestations of disease.

## Methods

Cases with detection of *Campylobacter* in any clinical isolate at the University Hospital Basel within the time period from April 2015 to January 2017 were eligible for this observational case-control study. A total of 119 consecutive *C. jejuni* and 15 *C. coli* bacteria were isolated. Coinfections were excluded. Rare *Campylobacter* species such as *C. fetus* (*n* = 2), *C. upsaliensis* (n = 2), and *C. concisus* (*n* = 1) were also detected, but subsequently excluded from the analysis due to the small sample size. Additionally, 22 *Campylobacter* isolates were detected at the University Children’s Hospital Basel during the same observation period. Due to small sample size, these data were only analysed descriptively and not included in the case-control study.

### Patients and data collection

The study has been approved by the local ethical review board (EKNZ, approval number 2016–01183). The medical records of the included patients were retrospectively reviewed. Data extraction was performed blinded for T6SS status. Hospitalised cases included in this study were admitted via the emergency unit. Outpatients were most commonly treated in the emergency unit and in some cases monitored for a maximum of one night.

The following data were systematically collected: demographic characteristics, medical and travel history as pertaining to the present infection. In addition, clinical characteristics at presentation, characteristics of diarrhoeal disease, and laboratory results at presentation, as well as the requirement for antibiotic treatment, were documented. Underlying diseases were recorded according to the Charlson Comorbidity Index [18–21]. Regarding treatment, fluid substitution and antibiotic therapy (including duration and application) were recorded. Information on the need for and duration of hospitalisation, need for ICU-stay and in-hospital mortality were extracted. Microbiological data included identification to the species level and the antibiotic susceptibilities to erythromycin and ciprofloxacin (μg/mL).

The following categorical factors were defined as primary clinical endpoints: need for hospital admission, antibiotic therapy, intensive care unit (ICU) stay, development of bacteraemia, and in-hospital mortality.

### Isolate growth and identification

All routine clinical microbiology was performed in an ISO/IEC 17025 accredited laboratory. Stool samples were cultured in microaerobic conditions (5% O2, 10% CO2 and 85% N2) on Campylosel agar plates (bioMérieux, Marci I’Étoile, France) at 41 °C for 48 h. Bacterial isolates were identified using Matrix Assisted Laser Desorption Ionization – Time of Flight mass spectrometry using methods described by Bessède et al. [22] on a Microflex LT mass spectrometer and MALDI Biotyper Compass software v. 4.1.70 (Bruker Daltonics, Germany). The value of “Score” ≥ 2.0 was accepted as a measure for reliable species identification.

### Minimal inhibitory concentration (MIC) determination

MICs for erythromycin and ciprofloxacin of each isolate were determined by E-test (bioMérieux) and incubated under microaerobic conditions on MHF (Müller Hinton agar + 5% horse blood + 20 mg/l β-NAD) agar plates (bioMérieux) at 41 °C for 24 h. The interpretation of MICs was performed according to EUCAST guidelines (version 8.1; www.eucast.org).

### Type VI secretion system (T6SS) PCR detection

PCR detection of the *hcp* gene as a locus representing the T6SS in *C. jejuni* and *C. coli* isolates was performed [23]. We used *C. jejuni* subsp. *jejuni* strains from ATCC (Ref. 33560 and 43431) as positive controls. Briefly, the DNA of cultured isolates was extracted using the DNA Bacterial card on the EZ1 Advanced XL automated extraction system (Qiagen, Hombrechtikon) using the manufacturer’s protocol following a digestion step using proteinase K performed for 10 min at 56 °C and 10 min at 95 °C. The PCR used the following primers: Hcp_F 5′-CAAGCGGTGCATCTACTGAA-3′, Hcp_R 5′-TAAGCTTTGCCCTCTCTCCA-3′ and as a control the *gltA* gene gltA_F 5′-GCCCAAAGCCCATCAAGCGGA-3′, gltA_R 5′-GCGCTTTGGGGTCATGCACA-3′. HotStarTaq (Qiagen) was used for the PCR with a melting temperature of 57 °C. PCR fragments (*hcp* 463 bp and *gltA* 142 bp) were detected on a 2200 TapeStation instrument (Agilent, Santa Clara, USA) using the D1000 ScreenTape assay (Agilent).

### Whole genome sequencing (WGS)

Isolate details are given in Additional file 1: Table S1. DNA was extracted as above. Library generation with Nextera XT (Illumina, Cambridge, UK) was followed by sequencing 2 × 300 bp on an Illumina Miseq platform. Downstream analysis was performed in CLC Genomics Workbench v 10.1.1 with read trimming and QC, followed by coverage determination by mapping against the genome of NCTC11168 (accession number NC002163). All genomes gave a mean coverage over 62x, up to 488x. The presence of the T6SS was determined by mapping against the well characterised T6SS locus in strain 414 (accession number CM000855, locus tags C414_000040086- C414_000040100) [13]. All isolates with reads mapping to under 25% of the gene cluster, also with mean coverage of the region lower than 1x, were considered as negative (*n* = 103); those mapping to over 86% of the locus were considered to be positive (*n* = 21); and those mapping to 26–41% of the locus considered as partial (n = 10). Read data generated for this study can be found in the European Nucleotide Archive (ENA, http://www.ebi.ac.uk/ena/) under project number PRJEB30092.

### Statistical methods

Descriptive analysis was performed with all data. For the analysis of the clinical impact of T6SS, only adult patients infected with *C. jejuni* were included into the statistical model to obtain a more homogenous cohort. Median and interquartile ranges are shown, unless otherwise indicated. Disease and host characteristics were compared in a univariate analysis between T6SS-positive (case) and negative strains (controls). For the calculation of adjusted odds ratios, a multivariable logistic regression model was constructed using variables that were statistically significantly associated with a positive T6SS status in the univariate analysis. Results with *p*-values < 0.05 (two-sided) were considered statistically significant.

## Results

### *C. jejuni* isolates are more often positive for T6SS than *C. coli*

From April 2015 to January 2017, we identified a total of 119 cases of *C. jejuni* infection and 15 cases of *C. coli* infection from an adult population. All cases were community acquired and no nosocomial infections occurred. Six of 119 *C. jejuni* strains (5.0%) were isolated from blood culture. In two patients with bacteraemia, stool culture was also positive for the same species.

All isolates from the adult population (*n* = 134) were tested for the presence of the T6SS, first by PCR targeting the *hcp* gene, and also by WGS. Data agreed in all cases. Overall, 21 isolates (15.7%) were found to be T6SS positive. Regarding species, 20/119 (16.8%) *C. jejuni* and 1/15 (6.7%) *C. coli* isolates were found to be T6SS positive. In 10 cases, partial mapping to the T6SS was observed, with reads mapping to the locus tags C414_000040089 and C414_000040100, but not to *hcp* (C414_000040085). Interestingly, mapping coverage to the T6SS in the positive isolates represented a relative coverage of 1–36% compared to mapping across the whole genome (Additional file 1: Table S1).

Additionally, in a paediatric population, 19/22 (86.4%) *C. jejuni* and 3/22 (13.6%) *C. coli* were isolated. The isolates were screened by PCR for the *hcp* gene locus, which was found in 3/19 (15.8%) *C. jejuni* strains. Due to the low frequencies of isolates from paediatric patients and the obvious expected differences in both cohorts, we focused our further analysis on bacterial isolates from adult patients. Additional file 2: Table S2 provides the clinical characteristics of children infected with *Campylobacter* spp.

### T6SS positive *C. jejuni* isolates are associated with resistance towards ciprofloxacin

All *Campylobacter* strains from the adult population were tested for erythromycin and ciprofloxacin susceptibility. Two of 119 *C. jejuni* (1.7%) and three of 15 *C. coli* (20.0%) had resistant MICs for erythromycin. 72 of 119 *C. jejuni* (60.5%) and 10 of 15 *C. coli* (66.7%) had resistant MICs for ciprofloxacin. All 20 T6SS-positive *C. jejuni* strains were susceptible to erythromycin, whereas 17 of 20 T6SS-positive *C. jejuni* strains (85.0%) were resistant to ciprofloxacin (Chi-squared, *p* = 0.01).

### Clinical data

Clinical characteristics of infection in adult patients with *C. coli* and *C. jejuni* are listed in Table 1. Infection with *C. jejuni* manifested in 117/119 cases, and in all 15 *C. coli* cases, as gastrointestinal disease with diarrhoea, often accompanied by abdominal pain (68.1 and 46.7%, respectively) and vomiting (28.6 and 20.0%, respectively). Patients also commonly reported fever (52.1 and 46.7%, respectively).

**Table 1:** Adult patient characteristics

Characteristics	Patients with *C. coli* infection	Patients with *C. jejuni* infection	All adult patients
	(*n* = 15)	(n = 119)	(n = 134)
Age (median years)	51 (26–80)	57 (33–74)	55 (33–74)
Male gender	7 (46.7)	74 (62.2)	81 (60.4)
Hospitalised patients	7 (46.7)	73 (61.3)	80 (59.7)
Duration of hospitalisation (median days)	0 (0–6)	1 (0–5)	1 (0–5)
Fever history (yes)	7 (46.7)	62 (52.1)	69 (51.5)
Bloody diarrhoea (yes)	2 (13.3)	21 (17.6)	23 (17.2)
Antibiotic therapy (yes)	9 (60.0)	73 (61.3)	82 (61.2)
Leucocytes (×10^9 /l)	8.5 (5.0–12.1)	8.6 (5.9–11.2)	8.5 (5.9–11.3)
C-reactive protein (mg/l)	69 (41–186)	66 (29–129)	68 (31–133)

Data are median (IQR) or n (%)

In patients with *C. jejuni* infection, underlying diseases were present in 47.9%. Malignancies (including solid neoplasia, leukaemia and lymphoma) and diabetes mellitus type 2 were identified as the leading comorbidities. Eleven patients had an immunocompromised status due to a solid organ transplant, allogenic haematologic stem cell transplant (HSCT) or AIDS. The course of disease was uncomplicated in most cases. 61.3% of patients had to be hospitalised. Two patients were admitted to ICU, one bacteraemic patient died at the hospital. Table 2 compares hospitalised patients with patients receiving outpatient care. Hospitalised patients were older, showed a higher leucocyte count and higher CRP-values, which are all indicators of a more severely affected cohort. Creatinine clearance was lower in hospitalised patients.

**Table 2:** Comparisons between adult patients hospitalised and receiving outpatient care with *C. jejuni*

	Need for hospital admission	Out-patient treatment	*p*-value
	(*n* = 73)	(*n* = 46)	
Male gender	48 (65.8)	26 (56.5)	
Age (median years)	69 (49–81)	45 (27–63)	< 0.001
Leucocytes (×10^9 /l)	9.2 (6.7–11.9)	7.6 (5.6–10.2)	0.05
C-reactive protein (mg/l)	84 (39–171)	43 (10–87)	0.001
Creatinine clearance (ml/min)	68 (41–95)	88 (71–108)	0.004

Data are median (IQR) or n (%). *P* values ≤0.05 are shown

### Comparisons between patients infected with *C. jejuni* harbouring and not harbouring T6SS

Due to the low frequency of T6SS-positive *C. coli* strains, only *C. jejuni* were included in the final statistical model. We analysed the *C. jejuni* adult cohort (*n* = 119) with respect to the T6SS status of each isolate, to ascertain possible associations with the virulence factor. Table 3 shows a comparison of patients infected with T6SS-positive versus T6SS-negative *C. jejuni* isolates, focusing on patient characteristics and clinical course of disease. None of the defined clinical endpoints was associated either with T6SS-positive or negative *C. jejuni* isolates: 60.0% of patients with T6SS-positive isolates and 61.6% with T6SS-negative isolates were admitted to the hospital, respectively. Antibiotic therapy was administered to 65.0% of patients infected with T6SS-positive and in 60.6% of patients with T6SS-negative isolates. One of 20 patients with a T6SS-positive isolate and one of 99 patients with a T6SS-negative strain was admitted to ICU. Of note, bacteraemia was present only in patients infected with T6SS-negative strains (*n* = 6), leading to a single patient death in hospital.

**Table 3:** Comparisons between adult patients infected with T6SS-positive and T6SS-negative *C. jejuni*

	T6SS-positive	T6SS-negative	*p*-value
	(*n* = 20)	(*n* = 99)	
Baseline characteristics			
Age (median years)	58 (46–77)	57 (31–74)	n.s.
Male gender	13 (65.0)	61 (61.6)	n.s.
Hospitalised patients	12 (60.0)	61 (61.6)	n.s.
Duration of hospital stay (median days)	3 (0–5)	1 (0–5)	n.s.
ICU stay (yes)	1 (5.0)	1 (1.0)	n.s.
Comorbidities			
Charlson Comorbidity Index	2 (1–5)	0 (0–1)	< 0.001
Solid organ transplant	4 (20.0)	3 (3.0)	0.015
Immunosuppressant therapy	6 (30.0)	9 (9.1)	0.019
Clinical characteristics			
Bacteraemia	0 (0.0)	6 (6.1)	n.s.
Fever history (yes)	6 (30.0)	56 (56.6)	0.048
Bloody diarrhoea (yes)	2 (10.0)	19 (19.2)	n.s.
Duration of diarrhoea (days)	6 (4–10)	6 (4–9)	n.s.
Leucocytes (× 10^9^/l)	8.5 (6.1–12.1)	8.7 (5.9–11.1)	n.s.
Antibiotic therapy (yes)	13 (65.0)	60 (60.6)	n.s.

Data are median (IQR) or n (%). *P* values ≤0.05 are shown. Only *p*-values of significant results were shown. n.s. non-significant

Patients infected with T6SS-positive *C. jejuni* isolates had a higher score in the Charlson Comorbidity Index, were more likely to have had a solid organ transplant, or to be under immunosuppressive therapy as compared to patients infected with T6SS-negative isolates. A higher comorbidity score remained associated with infection with a T6SS-positive strain in multivariable analysis including the CCI, solid organ transplants and immunosuppressive therapy in the final regression model (Table 4).

**Table 4:** Uni- and multivariable logistic regression models

	Crude			Adjusted^a^		
	OR	95%CI	*p*-value	OR	95%CI	*p*-value
Charlson Comorbidity Index	1.71	1.30–2.25	< 0.001	1.65	1.25–2.18	< 0.001
Solid organ transplant	8.08	1.65–39.54	0.010	1.82	0.19–17.83	0.608
Immunosuppression	4.33	1.34–14.05	0.015	2.41	0.41–14.10	0.328
Antacids	2.6	0.98–6.89	0.055			

^a^Charlson Comorbidity Index, solid organ transplant and immunosuppressants were included in the final regression model

*OR* odds ratio, *95%CI* 95% confidence interval

## Discussion

This study adds to the growing amount of research on the bacterial T6SS providing insight into its occurrence in clinical *Campylobacter* spp. and examining its role in human infection.

While 15.7% of the isolates from the adult population were positive for the complete T6SS locus, a proportion also carry partial T6SS gene clusters, as has previously been noted [15, 24]. These were not identified by PCR primers targeting *hcp*, meaning that the PCR of *hcp* is a valuable marker for a functional T6SS [15]. That mean coverage of the T6SS is lower than that across the genome may be a sequencing artefact relating to the relatively low %G + C content of this locus, or may indicate that the locus is lost from some bacteria during culture.

The clinical characteristics of infection with *Campylobacter* spp. in the study population are similar to those previously described by various authors [1, 6]. Infection with *C. jejuni* and *C. coli* were clinically indistinguishable: both manifested as acute gastroenteritis with a similar distribution in both species causing vomiting, abdominal pain, bloody diarrhoea and fever history. Complications arose in very few cases, with a single death attributable to invasive campylobacteriosis. In view of epidemiologic studies reporting a peak incidence of *Campylobacter* infection in toddlers and young adults [1, 25, 26], the median age of 55 in patients in the present study seems relatively high. However, Schmutz et al. [27] describe a trend towards a higher median age in *Campylobacter* infections in Switzerland. A possible selection bias towards an older and sicker population remains: 59.7% of all diagnosed patients required hospital admission and antibiotic therapy was prescribed in 61.2% of cases. Furthermore, 5.0% of patients infected with *C. jejuni* had a positive blood culture, which is unusually high considering population-based studies from Denmark [28] and the USA [29]. These have described an incidence of *Campylobacter* bacteraemia of less than 1% of all infections. Besides the aforementioned selection bias, this discrepancy may also reflect the higher number of automatic blood cultures performed in a hospital setting.

Various studies have investigated the epidemiology of T6SS in *C. jejuni*. The T6SS seems to be more prevalent in isolates from Asia and Egypt than Europe, with 60.6% of human isolates T6SS-positive in Vietnam [15], 33.3% in Thailand [15] and 57.6% in a large Egyptian paediatric cohort [30]. European studies have shown a prevalence of 2.6% in human isolates from the UK [15] and 14% in chicken isolates from Spain [24]. The results of the present study are in accordance with these studies in that they uphold the large difference between Asian and European strains. With a T6SS-prevalence of 16.8% in *C. jejuni* the results are close to those from Spain [24], although their strains were obtained from poultry and sewerage water. Bleumink-Pluym et al. also identified a T6SS-prevalence of about 10% in strains from various sources such as human, chicken, swine, and cattle [14].

The reported prevalence of T6SS in *C. coli* reaches from 18.0% in isolates from a paediatric cohort [30] to 56.1% in isolates from retail chicken [23]. Corcionivoschi et al. [23] found a much higher percentage of T6SS-positive *C. coli* in comparison with *C. jejuni*. As they point out, only a small percentage of *Campylobacter* infections in humans are caused by *C. coli*, whereas the prevalence of *C. coli* and *C. jejuni* is almost equal in their study [23]. The present study identified only one functional T6SS among 15 *C. coli* isolates. The low prevalence of 6.7% may be due to small sample size, however: Sainato et al. [30] also recorded a relatively low frequency of T6SS in *C. coli* (18%) in comparison to *C. jejuni* (57.6%) in clinical isolates.

Our comparison between patients with T6SS-positive and -negative *C. jejuni* isolates did not reveal a significant difference in the clinical manifestations and the course of disease. All variables defined as clinical endpoints (e.g. hospital admission, antibiotic therapy) showed a similar distribution in both groups. These findings agree with a recently published study examining 176 cases of infection with *C. jejuni* and 126 cases of infection with *C. coli* in a paediatric cohort from Egypt concerning the presence of the *hcp* gene [30]. The clinical symptoms did not differ in severity between T6SS-positive and –negative *Campylobacter* strains [30]. This stands in contrast to the findings of Harrison et al. [15] of a positive association of T6SS-presence in *C. jejuni* with bloody diarrhoea. Bleumink-Pluym et al. [14] noted that 50% of their T6SS-positive strains were isolated from blood, also suggesting an association with more severe disease. The studies were performed with 36 and 8 *C. jejuni* strains, respectively, making it possible that the dissimilarity in findings is due to small sample size and potentially other virulence factors.

We found a surprisingly stable association (*p* < 0.001) with T6SS-positive strains for a higher score in the Charlson Comorbidity Index, ascertained also in a multivariable model. Cancerous diseases and diabetes mellitus type 2 were identified as the leading comorbidities. These and other diseases assessed by the CCI show an immunosuppressing effect in patients. It seems that patients with a higher morbidity, but also with an immunocompromised status are more susceptible to *C. jejuni* strains harbouring a T6SS. The association of T6SS-positive strains with allogenic haematopoietic stem cell transplantation (HSCT), solid organ transplant and immunosuppressant therapy (though not independently) supports this hypothesis. In a study of *Acinetobacter baummanii* isolates, Kim et al. [31] also found an association between infection with T6SS-positive strains and HSCT and immunosuppressant therapy. Our results suggest a possible contribution of the T6SS to infection of an immunocompromised and sickly host. The mechanism, however, is not clear and should be further investigated. The identified association may, however, be influenced by selection bias towards an older and more sickly population.

The rates of antibiotic resistance identified in the present study are similar to those reported by other authors [6, 32] with macrolide resistance higher in *C. coli* (20.0%) than in *C. jejuni* (1.7%) and generally high resistance rates for ciprofloxacin: 61.2% of all *Campylobacter* isolates. Analysis of T6SS-positive *C. jejuni* strains showed a significantly higher resistance rate for ciprofloxacin. A possible explanation for this association may be found in a correlation with clonal complexes analysed by multi-locus sequencing typing. Kittl et al. [3], Collado et al. [33] and Kovac et al. [34] examined the relationship between ciprofloxacin-resistance and sequence types in *Campylobacter jejuni* showing a significant association with clonal complexes ST-21 [3, 33, 34], ST-353 [33, 34], ST-48 [33] and ST-464 [3]. Kittl et al. noted that the correlation was independent from the host, suggesting that certain STs may develop more easily quinolone resistance [3]. Harrison et al. [15] found a correlation between the T6SS and clonal complex ST-353. It seems that clonal complexes showing higher resistance for ciprofloxacin may also be those that are associated with carrying the T6SS.

Our study has some important limitations: the overall sample size is relatively small and for the statistical analysis we have only focused on *C. jejuni* isolates from adult patients (*n* = 119). Also, a relative high proportion of patients was hospitalised, which could reflect a more severely ill and immunosuppressed patient population at a tertiary hospital setting. Finally, we found only 20 *C. jejuni* isolates with a T6SS locus.

## Conclusions

Overall, our study does not provide evidence that the T6SS is associated with a more severe clinical course in *Campylobacter jejuni*. Interestingly, T6SS positive isolates are more commonly found in immunocompromised patients: an observation which merits further investigation.

## Additional files

Additional file 1: **Table S1.** Isolates and genome data. Description of data: List of analysed *Campylobacter* isolates with WGS and PCR results on the presence of T6SS. (XLSX 21 kb)

Additional file 2: **Table S2.** Paediatric patient characteristics. Description of data: Table with descriptive statistics on a paediatric cohort of 22 patients. (DOCX 13 kb)

## Abbreviations

CCI: Charlson Comorbidity Index
EKNZ: Ethikkommission Nordwest- und Zentralschweiz
*hcp*: Haemolysin coregulated protein
HSCT: Haematologic stem cell transplant
ICU: Intensive care unit
PCR: Polymerase chain reaction
T6SS: Type VI secretion system
WGS: Whole genome sequencing
