# Supplementary material for: Clinical impact of the type VI secretion system on virulence of Campylobacter species during infection
Source: BMC Infect Dis. 2019 Mar 7;19:237. doi: 10.1186/s12879-019-3858-x (PMC6407262; doi:10.1186/s12879-019-3858-x)
Supplement: Supplementary file 2 — Table S2. Paediatric patient characteristics. Description of data: Table with descriptive statistics on a paediatric cohort of 22 patients. (DOCX 13 kb) [file 12879_2019_3858_MOESM2_ESM.docx]

| **Supplementary Table 2.** Paediatric patient characteristics. | | | |
| --- | --- | --- | --- |
| **Characteristics** | **Patients with *C. coli* infection** (n=3) | **Patients with *C. jejuni* infection** (n=19) | **All paediatric patients** (n=22) |
| Age (median years) | 13.1 (7-14.9) | 7.6 (2.4-13.8) | 8.1 (1.9-13.9) |
| Male gender | 2 (66.7) | 14 (73.7) | 16 (72.7) |
| Hospitalised patients | 1 (33.3) | 8 (42.1) | 9 (40.9) |
| Duration of hospitalisation (median days) | 3 | 2 (2-3) | 2 (2-3) |
| Fever history (yes) | 3 (100) | 19 (100) | 22 (100) |
| Bloody diarrhoea (yes) | 2 (66.7) | 12 (63.2) | 14 (70) |
| Antibiotic therapy (yes) | 0 | 3 (15.8) | 3 (13.6) |
| Leucocytes (x10^9 /l) | 9.6 (9.5-13.8) | 10.7(7.9-13.6) | 10.6 (8.2-13.6) |
| C-reactive Protein | 40.0 (26.0-85.5) | 55.8 (29.5-97.9) | 40.0 (29.0-103.3) |
| T6SS (hcp+) | 0 | 3 (15.8) | 3 (13.6) |
| Data are median (IQR) or n (%). | | | |
